# The Epigenomic Impact of Quantum Dots: Emerging Biosensors and Potential Disruptors

**DOI:** 10.3390/epigenomes9040050

**Published:** 2025-12-08

**Authors:** Abhishu Chand, Kyoungtae Kim

**Affiliations:** Department of Biology, Missouri State University, 901 S National, Springfield, MO 65897, USA; ac43s@missouristate.edu

**Keywords:** quantum dots, epigenetics, dual role, biosensor, epigenetic disruptor

## Abstract

Quantum dots (QDs) have emerged as powerful tools in biomedical applications due to their unique optical and fluorescent properties which enable highly sensitive and multiplexed detection of biomolecules. Particularly in the field of epigenetic research, QDs are utilized as biosensors for monitoring changes in DNA methylation, microRNA (miRNA) expression, and histone modifications, providing a viable alternative to conventional assays. However, increasing evidence also suggests that QDs act as an epigenetic disruptor, altering epigenetic mechanisms and downstream cellular processes. This dual role raises important questions about the safety, reliability, and translational potential of QDs in clinical usage. Therefore, in this commentary we critically evaluate the advances of QD-based epigenetic sensing platforms while also providing insights into QD-based epigenetic dysregulation. We further discuss the current limitations and provide future directions to gain a better understanding of how QDs function to bridge the gap between their diagnostic potential and clinical integration.

## 1. Introduction

**Figure 1:**
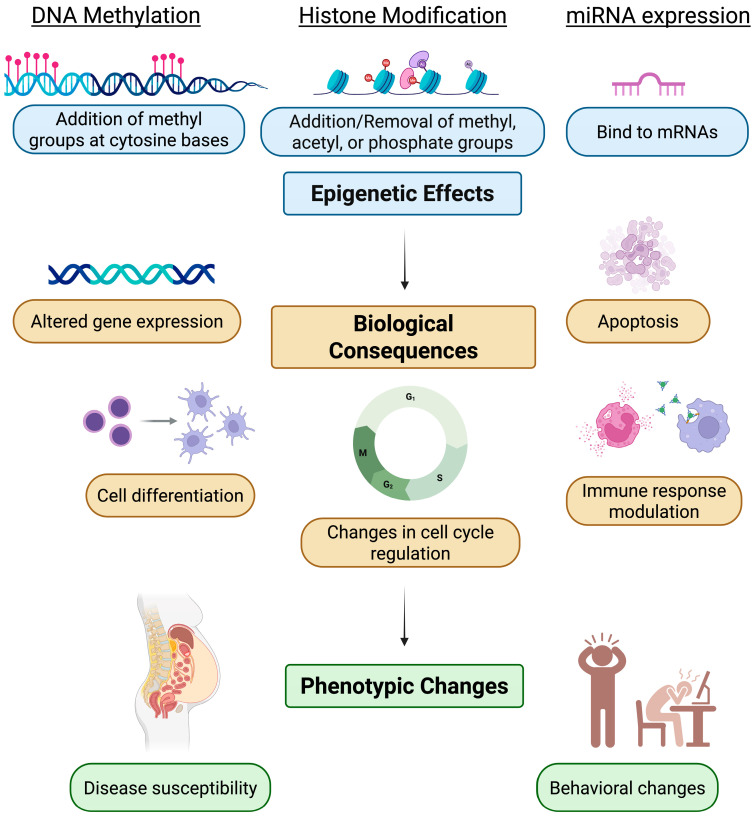
Schematic model demonstrating epigenetic effects, their biological consequences, and impact on phenotypic changes (created in BioRender. Kim, K. (2025) https://BioRender.com/7qjxzkv accessed on 29 September 2025).

Traditional genetics does not account for the changes in gene expression without alteration in DNA sequences. As such, the term epigenetics was coined by Conrad Waddington in 1942, where his understanding of epigenetics can be regarded as an attempt to explain the interactions between the environment and genes for the development of certain phenotypes [1,2]. Today, in biology, “epi” is a prefix that means “above” and in this instance implies above the genome, examining how environmental and external factors can influence gene expression without altering the underlying DNA sequences [3,4]. Epigenetic mechanisms involve DNA methylation, histone modification, and non-coding RNA-mediated processes [5,6], which lead to alteration of the gene expression mainly at the level of transcription (Figure 1), with indirect effects on translation as well [7,8]. DNA methylation and histone modification control gene expression by regulating the accessibility of DNA to the transcription machinery. The addition of methyl groups at cytosine bases of DNA can silence genes by preventing transcription factors from binding to DNA or by recruiting proteins that condense chromatin [9,10,11]. Similarly, histones can be modified by adding or removing functional groups like methyl, acetyl, or phosphate groups (Figure 1) to regulate gene expression [12]. In addition, non-coding RNAs such as microRNAs (miRNAs) influence gene expression by binding to target messenger RNAs (mRNAs), resulting in mRNA degradation or translation inhibition. MiRNAs are also able to alter chromatin structure by directly binding to DNA sequences within or near gene promoters to modify histones and DNA methylation patterns [13,14,15].

In parallel to advances in molecular biology, the field of nanotechnology has given rise to novel tools for biomedical applications, consumer products, and environmental solutions. The widespread applications of nanoparticles (NPs) have resulted in extensive studies on their biological effects including genotoxicity [16,17,18] and influence on gene regulation via epigenetic pathways. Although the overlap of studies involving nanotechnology and epigenetics are relatively new, several studies have demonstrated the ability of NPs to alter DNA methylation patterns and dysregulate miRNA expression without altering the DNA sequence [19,20,21].

Among these NPs, quantum dots (QDs), a type of semi-conductor nanoparticle, first discovered by Alexey Ekimov and Alexei A. Onushchenko in 1981 [22], have been highly sought after for their unique optical and fluorescent properties. These properties are widely explored for drug delivery, tracking, and bioimaging [23,24,25,26,27]. As such, extensive research has been carried out to tap into QDs’ potential. While these studies substantially cover the biological effects of QDs on human health and the environment [28,29,30], very few of them discuss the epigenomic impact of these QDs, particularly focusing on long-term impact and heritable effects. Given their immense potential and increasing integration in biomedical applications, it is essential to study whether QDs may not only detect epigenetic alternations but also induce them. While QDs are mainly used as nanobiosensors to detect epigenetic changes [31,32,33], they themselves bear the risk of affecting and causing changes that may last several generations due to their reactivity and biological toxicity. This dual role as an epigenetic biosensor and disruptor warrants a more integrated approach to study the complexity of QD function and toxicity. Therefore, this commentary focuses on incorporating the current literature to critically analyze studies that present (1) QDs as tools for detection of epigenetic changes and (2) their capacity to cause epigenetic dysregulation. It further emphasizes the necessity for long-term multigenerational studies, not only to understand QDs’ biological consequences, but to address risk assessment and regulation, especially with the increasing prevalence of QDs in the biomedical field.

## 2. QDs as Biosensors for Epigenetic Process Detection

QDs share unique optical properties such as high photostability, size-tunable fluorescence, and broad excitation with narrow emission spectra [25,34] that enable their usage as nanobiosensors for detection of epigenetic mechanisms. These properties are well-suited for highly sensitive real-time monitoring of epigenetic processes like DNA methylation, histone modifications, and miRNA expression. Therefore, a few studies dealing with the QD-based detection of these epigenetic processes are discussed below.

### 2.1. QD-Based Biosensors for DNA Methylation

**Figure 2:**
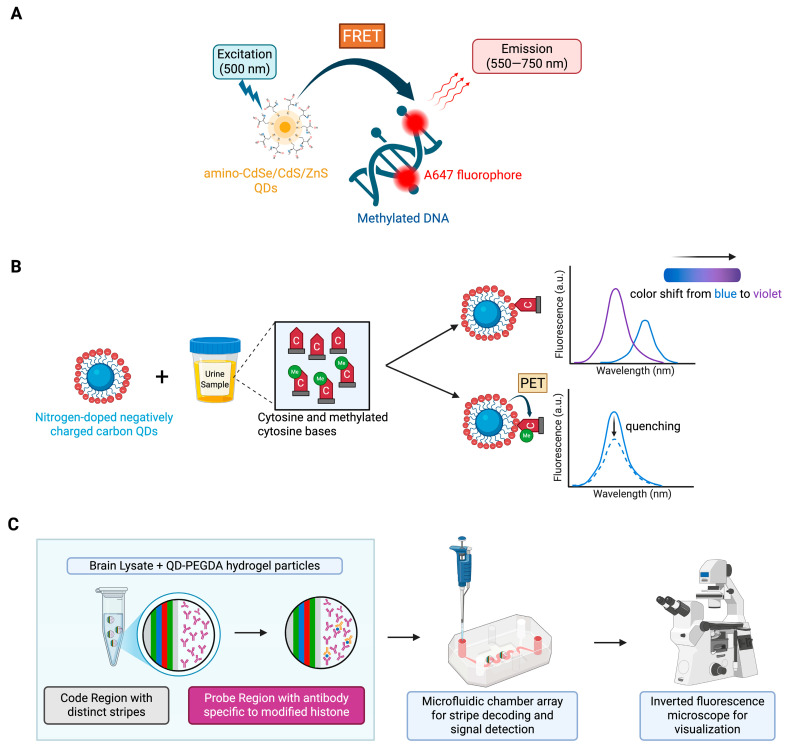
Illustrating QD-based epigenetic platforms. (**A**) QD-FRET system for detection of DNA methylation. Here, QDs act as energy donors to the A647 fluorophores which in return amplify the fluorescence (adapted from Ma et al. [35]). (**B**) Nitrogen-doped QDs for simultaneous detection of cytosine and 5-methylcytosine. The interactions between the nucleobases and CQDs result in fluorescence peak shift and quenching. The emission spectra were reproduced from Thonghleung et al. [36]. (**C**) QD-PEGDA hydrogel particles for multiplex histone modification detection. Each particle is associated with a distinct QD code and antibody-specific code region that are captured in a microfluidic chamber array and visualized in an inverted microscope. The structure of the particle was reproduced from Yeom et al. [37]. (Created in BioRender. Kim, K. (2025) https://BioRender.com/wyarldc accessed on 29 September 2025).

DNA methylation is a key epigenetic mechanism and one of the most well-characterized chemical modifications of the genome. It plays a crucial role in cell differentiation and development, and its patterns can be altered by environmental factors such as lifestyle, chemical exposure, and biological stressors, which collectively contribute to disease susceptibility (Figure 1). Consequently, QD-based fluorescence resonance energy transfer (FRET) systems have been utilized for detection of DNA methylation with high sensitivity. For example, in the system utilized by Ma et al., genomic DNA was treated with methylation-sensitive restriction enzymes that cleave unmethylated DNA at specific recognition sites, leaving the methylated sites intact. Next, the methylated DNA was amplified by polymerase chain reaction (PCR) with simultaneous incorporation of Alexa Fluor-647 (A647) fluorophores (Figure 2A). The PCR products were then mixed with QDs to induce FRET, and the DNA methylation levels were correlated with FRET intensity [35].

The authors compared the efficacy of this QD-FRET system with that of pyrosequencing, the gold standard for DNA methylation analysis. Although the quantitative methylation values showed some inconsistencies, the overall trend of DNA methylation in non-treated vs. cancer samples was similar between the two techniques. The inconsistencies were attributed to incomplete DNA digestion by the restriction enzyme. Although the study does not particularly mention the limit of detection (LOD), they carried out a receiver operating characteristic (ROC) analysis to assess the sensitivity and specificity of the system, where they found the QD-FRET system to show 95% specificity and sensitivity up to 90% for combined detection of methylated tumor suppressor genes (*PCDHGB6*, *HOXA9*, and *RASSF1A*). In comparison, a study carried out by Molano et al. using pyrosequencing on *EPB41L3*, another tumor suppressor gene, reported that the combined detection of methylation at CpG site 2 and 4 of *EPB41L3* had a sensitivity of 95.5% and specificity of 60% [38]. While the sensitivity and specificity can vary depending on the gene and target CpG sites, it can be observed that the efficacy of the QD-FRET system is comparable with other established techniques. Together, the proposed QD-FRET system provides an efficient and cost-effective approach for non-invasive detection of cancer via DNA methylation analysis. However, since the study did not provide a quantitative LOD and relied on relative FRET ratio measurements instead of absolute methylation percentages, it is difficult to draw direct comparisons with established techniques such as pyrosequencing and methylation-sensitive high-resolution melting (MS-HRM).

In addition to QD-FRET systems, researchers have also focused on developing safer and more biocompatible alternatives. For example, a novel dual-responsive nitrogen-doped carbon QD (CQD) fluorescent sensor was developed by Thonghleung et al. for simultaneous detection of cytosine and 5-methylcytosine (5-mC) in urine samples. The detection platform is driven by the interactions between CQDs and nucleobases (cytosine), which results in a fluorescence color shift from blue to violet as well as an increase in fluorescence intensity (Figure 2B). On the other hand, the presence of 5-mC results in fluorescence quenching of the CQDs caused by a photoinduced electron transfer [36]. These contrasting behaviors enable simultaneous detection, which might be useful to detect the whole-body epigenetic status, as alterations in 5-mC levels have been associated with various biological processes and diseases, including cancer and Alzheimer’s [39,40].

The detection limits of 43.4 μM for cytosine and 74.4 μM for 5-mC are sufficient for urinary biomarker analysis but are not expected to be as sensitive as liquid chromatography–mass spectrometry (LC-MS), which typically achieves LODs in the low µM to nM range. A direct comparison between the two systems would have strengthened the study by analyzing the sensor’s performance. Moreover, clinical validation by testing patient samples with known methylation disorders or diseases would have provided the relevance of the sensor in biomedical settings. While this novel technology offers greater simplicity, speed, and affordability, it lacks locus-specific resolution compared to current technologies such as bisulfite sequencing. Nevertheless, the study lays a strong foundation to integrate a dual fluorescent sensor into clinical and diagnostic workflows as a complementary tool to existing technologies, ideal for epigenetic screening and routine monitoring purposes.

### 2.2. QD-Based Biosensors for Histone Modification

Histone modifications are another key epigenetic mechanism that regulate gene expression by altering the chromatin structure. As mentioned earlier, although these modifications occur post-translationally, they influence transcriptional activity by controlling the accessibility of DNA to the transcription machinery. To study the impact of histone modifications in epigenetic analyses, QDs are incorporated with other nanoparticles to form hybrid sensors that enhance sensitivity, specificity, and multiplexing capabilities. In one such approach, Yeom et al. developed QD-encoded polyethylene glycol diacrylate (PEGDA) hydrogel microparticles (Figure 2C) for the direct multiplex detection of the following histone modifications: dimethylation of lysine 9 on histone H3 (2Me-H3K9), trimethylation of lysine 9 on histone H3 (3Me-H3K9), and acetylation of lysine 9 on histone H3 (Ac-H3K9) [37]. 2Me-H3K9 and 3Me-H3K9 are associated with repression, whereas Ac-H3K9 causes activation of gene expression. These markers were assessed by the authors to identify the epigenetic effects of chronic cocaine exposure to mice.

The QD-PEGDA platform consists of several microparticles, with each having a code region and a probe region. In the code region, QDs with a distinct emission wavelength (for example, blue, green, or red) are photochemically immobilized as stripes or codes into the microparticles via UV exposure. Each microparticle is embedded with a distinct color-coded QD stripe that allows for simultaneous imaging or identification of the markers using a single excitation wavelength. On the other hand, each microparticle has a probe region that is functionalized with antibodies specific to the target histone modification (Figure 2C). This distinct coding of QDs and specific antibodies onto each microparticle allows for the multiplex detection of histones.

Upon incubation with nuclear lysates, the histones bind to their specific capture antibodies. Next, a reporter antibody is added, followed by streptavidin-phycoerythrin (SA-PE) to bind to the reporter antibody and produce fluorescent signals. Finally, the microparticle system is loaded into a microfluidic chamber and imaged with an inverted fluorescence microscope [37]. It is important to note that in this system, QDs do not directly sense the histone modifications, but rather enable the multiplexed identification of histone modification by serving as a stable and distinguishable fluorescent barcode.

This method of detection achieved comparable results in its ability to detect changes in histone modification when compared with Western blot, a conventional method, utilizing only 1 µg of protein sample, which was 15 times less than the protein sample size used for Western blot. Although sensitive, the changes in ratiometric intensity between the histone modifications and the control protein were smaller (5%) compared to that of Western blot (20%). This difference in intensity was attributed to the lower amount of protein used and/or the absence of protein separation by size in the QD-PEGDA hydrogel microparticle system. Additionally, the study does not mention whether it used metal-based or metal-free QDs in its system, leaving uncertainty over the cytotoxicity of QDs.

### 2.3. QD-Based Biosensors for miRNA Expression

Among the major epigenetic mechanisms, miRNAs are considered the least traditional because they regulate gene expression post-transcriptionally at the RNA level. Additionally, miRNA expression itself is also influenced by other epigenetic mechanisms, thereby making them a complex and interconnected regulatory component. One of the earliest studies linking QDs and miRNA detection appeared in 2011, when Zhang et al. developed a QD-FRET nanosensor for rapid and sensitive detection of let-7a miRNA using a two-stage exponential amplification reaction (EXPAR) [41]. The first stage of EXPAR amplified miRNA, while the second stage converted the miRNA into reporter oligonucleotides that formed a sandwiched hybrid with biotinylated capture probes and Cy5-labeled reporter probes. These probes helped the hybrid interact with QDs, where QDs functioned as FRET donors for miRNA detection. Thereafter, the nanosensor achieved a remarkable detection limit of 0.1 aM. To test the specificity, they analyzed the let-7 miRNA members (let-7a, let-7b, and let-7c) using the let-7a specific templates and compared the Cy5 fluorescent burst counts. They defined a burst as a peak in the dataset that exceeded a preset threshold. Despite a single nucleotide difference between let-7a and let-7c, the Cy5 burst counts had an 8-fold difference, showing the high specificity of the system.

In a recent study, Hosseini et al. (2025) developed a fluorescence-based biosensor to detect let-7a miRNA using a FRET system combined with hybridization chain reaction (HCR) [42]. They synthesized a AuNPs@CdS QD system and functionalized them with molecular beacon oligonucleotides. Upon the introduction of the target miRNA, HCR was initiated and triggered FRET between AuNPs and CdS QDs to provide fluorescence quenching corresponding to the let-7a concentration. The biosensor achieved a LOD of 2 pM within a linear range of 10–45 pM and showed high specificity, as it was able to distinguish let-7a from other closely related miRNAs (let-7b, let-7c, miRNA-155, and miRNA-141). Moreover, a mixed sample containing the target and other oligonucleotides exhibited fluorescence intensity similar to the target-only sample, further showing the system’s specificity [42].

Both these studies by Zhang et al. and Hosseini et al. aim to detect let-7a miRNA through nucleic acid amplification combined with a QD-FRET system [41,42]. Zhang et al. developed an ultrasensitive enzymatic EXPAR-QD-FRET-based system capable of differentiating between a single nucleotide [41]. Comparatively, Hosseini et al. simplified the concept by removing the use of enzymes by using HCR and FRET between AuNPs and CdS QDs to detect let-7a at the pM level [42]. Although its sensitivity is several orders of magnitude lower, this method is cheaper and easier to implement. Therefore, while Zhang et al.’s QD-FRET system laid the foundation for ultrasensitive miRNA detection in biological applications, Hosseini et al.’s system offers a practical balance between analytical performance and scalability, indicating progress towards real-world feasibility [41,42].

### 2.4. Discussion on the Usability of QDs as a Biosensor of Epigenomics

Compared to traditional epigenetic modification detection methods such as bisulfite sequencing or chromatin immunoprecipitation sequencing (ChIP-seq), QD-based biosensors offer several quantitative advantages like higher sensitivity, faster analysis, and the potential for real-time measurements. QD-based assays are also said to be more specific and reduce the likelihood of false positive results when paired with methylation-sensitive restriction enzymes or target-specific probes [35]. But false positives are still not completely unavoidable as they might occur due to partial DNA digestion by the restriction enzymes. Furthermore, the brightness and tunable emission of QDs provides a platform for multiplex detection, allowing simultaneous monitoring of several methylation sites. In contrast, traditional techniques like methylation-specific PCR and standard bisulfite sequencing focus on analyzing methylation patterns at a specific locus [43] unless extended to advanced platforms like whole-genome bisulfite sequencing (WGBS), multiplex ligation-dependent probe amplification (MLPA), and multiplex surface-enhanced Raman scattering immunoassay. These techniques analyze multiple sites simultaneously but are often resource-intensive with advanced computational requirements and significant costs, which make them less accessible for routine use [44,45,46].

Additionally, while standard bisulfite sequencing is a powerful tool for DNA methylation analysis, bisulfite treatment involves harsh conditions like high temperature and low pH that can cause degradation of DNA and cannot differentiate between 5-mC and 5-hydroxymethylcytosine (5-hmC), as both are resistant to the bisulfite conversion. While specialized techniques like oxidative bisulfite sequencing (OxBS-seq) or TET-assisted bisulfite sequencing (TAB-seq) can overcome this limitation, they further add to the cost and complexity [47]. In comparison, QD-based biosensors offer a convenient and efficient method to measure gene-specific 5-hmC modifications (Table 1), eliminating the need for 5-hmC-specific antibodies or radioactive labels.

These advantages along with the above-related studies emphasize the expanding role of QDs in epigenetic diagnostics, offering a powerful alternative to conventional analysis methods. However, several challenges must be addressed for their widespread adoption as next-generation diagnostic tools. Foremost, the potential cytotoxicity and environmental hazard presented by QDs raises major biocompatibility and disposal concerns. To address these concerns, researchers are developing and utilizing metal-free QDs based on carbon that exhibit lower toxicity, if at all, and significantly improve environmental compatibility [53,54,55,56].

Next, in biological environments, probe fluorescence instability can compromise the detection accuracy, which is caused by the protonation of functional groups in acidic environments resulting in detachment from the QD surface [57,58]. A way to mitigate this could be utilizing pH-resistant linkers that remain stable over a wide pH range or covalent surface modifications to remove the pH-sensitive groups for stable fluorescence in both physiological and acidic conditions. Additionally, maintaining the probe quality and performance requires precise control over QD synthesis and surface modification, which can be challenging and result in batch variation.

The signal quantification utilized by the QD-FRET system can also be influenced by nonspecific interactions that add background signals and reduce the system specificity [59]. This challenge can be mitigated by optimizing surface chemistry through zwitterionic coating, which minimizes protein adsorption and hence reduces nonspecific binding. Another viable option could be utilizing a dual FRET system where two distinct FRET pairs confirm the target-specific binding, reducing the likelihood of false positives.

Crucially, the lack of standardized protocol makes it extremely difficult in terms of reproducibility, cross-study comparisons, and translation into clinical settings. The establishment of standardized guidelines is pivotal to ensure quality control for reliability, regulatory approval, and adoption of QD-based biosensors in epigenetic diagnostics. These challenges collectively highlight the need for system optimization and further research to fully harness their potential for use as versatile, novel tools in biomedical sensing applications.

## 3. Epigenetic Changes Caused by QDs

Despite the aforementioned promising potential of QDs and their usage in biomedical applications, there have been major concerns regarding the potential long-term health effects due to increasing evidence on QD toxicity. Correspondingly, an emerging area of interest is the ability of QDs to induce epigenetic changes by dysregulating DNA methylation, histone modifications, and miRNA expression. These alterations can cause acute changes in cell function and also have transgenerational toxic effects. While limited, studies have been carried out to understand how QDs influence the epigenetic mechanisms, as discussed below. A summary of the epigenetic alterations reported across these studies is provided in Table 2.

### 3.1. General Outline of Epigenetic Research Combined with QD-Induced Changes in Histone Modifications

Epigenetic research generally utilizes a combination of molecular techniques to analyze the effects of QDs on gene expression without altering the DNA sequence. These techniques focus on analyzing DNA methylation patterns, identifying histone modifications and miRNA expression to track changes and compare between non-treated control cells and cells treated with QDs. For example, modification of a histone can be detected by performing a Western blot with antibodies that specifically bind to the modified histone. In one such study carried out by Choi et al. in 2008, human breast epithelial cancer cells (MCF-7) treated with cadmium telluride (CdTe) QDs showed a significant decrease in acetylated histone 3 (Ac-H3) levels [60]. Ac-H3 is associated with transcriptional activation.

Building on these molecular findings, the epigenetic changes can be related to functional outcomes by assessing the downstream effects on cell viability utilizing cytotoxic assays such as XTT, trypan blue, and LDH assay. Additionally, the genes involved in these changes can be evaluated by carrying out transcriptomic analysis by RNA-sequencing, microarrays, or a more specific method like RT-PCR. In the same study by Choi et al., a trypan blue assay showed a decrease in cell survival upon QD treatment. Following this, they also carried out RT-PCR to identify the upregulation and downregulation of pro-apoptotic and anti-apoptotic genes, respectively, indicating the activation of the p53 pathway by QD treatment.

Furthermore, another key approach in epigenetic research includes studying whether these changes can be reversed by modulators like histone deacetylase (HDAC) inhibitors. HDACs help remove acetyl groups from the lysine residues on histone proteins. This causes histones to have a more positive charge, causing it to bind more tightly to the DNA and repressing transcription. Reversibility studies further help to establish that the changes are indeed epigenetic rather than the result of DNA damage or mutation, and they also help in understanding the underlying mechanistic pathways. Choi et al. also demonstrated the reversible aspect of epigenetic changes by partially restoring the activity of Ac-H3 (shown by Western blot) using an HDAC inhibitor, trichostatin A (TSA). Overall, this multi-step approach for detection of epigenetic marks, functional consequences, gene expression, and reversibility evaluation represent a general strategy adopted by researchers in nanoparticle–epigenetic toxicology studies.

**Figure 3:**
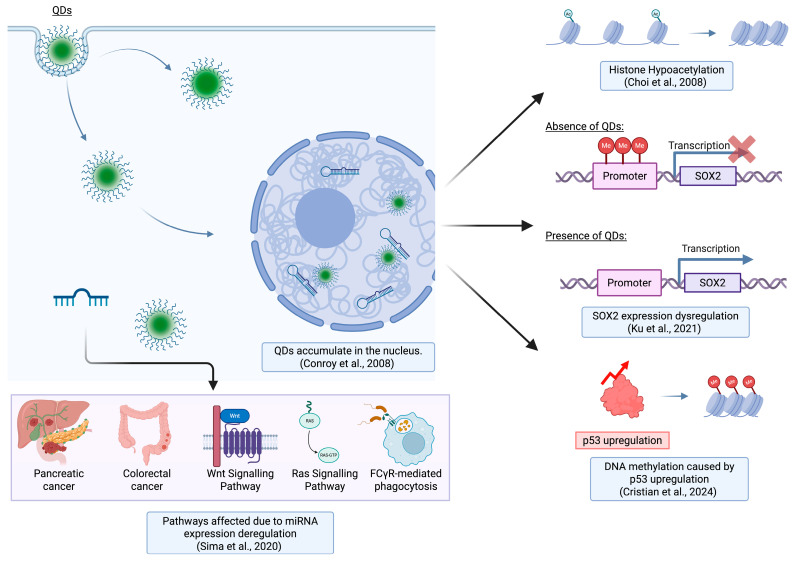
An illustration representing the epigenetic disruption caused by QDs. QDs alter miRNA expression, DNA methylation patterns, and modify histones, which affects key regulatory pathways and dysregulates gene expression. Adapted from Choi et al. [60], Conroy et al. [61], Sima et al. [63], Ku et al. [64], and Cristian et al. [65] (Created in BioRender. Kim, K. (2025) https://BioRender.com/g669d1u accessed on 29 September 2025).

Overall, this study by Choi et al. provided a strong foundation and highlights the importance and emergence of nanoepigenetics. The study focuses on the epigenetic (histone hypoacetylation) (Figure 3) and genotoxic (increased cell death) effects caused by QDs. Although an epigenetic mechanism is considered, the study does not observe whether these changes are heritable over generations. Additionally, the effects of these QDs are specific to the MCF-7 cell line only and studies should be carried out on other non-cancerous cell lines to gain more comprehensive insights on the epigenetic effects of QDs on healthy cells. Along with using electron microscopy to visualize nuclear changes, Western blot to look at histone modification, and TSA to look at HDAC activity, the epigenomic changes induced by QDs should be analyzed using techniques such as ChIP-seq and/or WGBS. ChIP-seq helps in identifying DNA binding regions and histone modification of specific proteins across the entire genome, whereas whole-genome bisulfite sequencing helps to identify DNA methylation patterns. While Western blot is useful to determine the presence of proteins, it is a semi quantitative approach as it relies on comparing the relative intensity of the protein bands, which can be subjective, especially when intensity differences are subtle. In contrast, WGBS is a highly quantitative method that measures the percentage of methylated cytosines at each CpG site across the genome. Moreover, Western blot also lacks detail at the genomic level but ChIP-seq and WGBS enable locus-specific and genome-wide profiling of epigenetic markers. These sequencing-based approaches result in precise mapping of histone modifications and DNA methylation patterns.

Another study carried out by Conroy et al. in 2008 examined the interactions of negatively charged CdTe QDs with nuclear components (RNA, DNA, and histones) in THP-1 cells, a human monocytic leukemia cell line [61]. Using fluorescent lifetime imaging (FLIM), they found that the QDs accumulated in the nucleus (Figure 3) and exhibited shorter lifetimes compared to those associated with the cell membrane. The reduced lifetime was attributed to QDs binding with nuclear biopolymers, suggesting that cellular localization affects QDs’ stability and interactions [61].

Next, using the dot-blot technique, they identified that QDs bind to core histones and nuclear lysate but not RNA or DNA. This was likely due to the study using negatively charged QDs, resulting in electrostatic repulsion with RNA and DNA. The QD–histone binding was further validated using absorption and photoluminescence (PL) spectra, where interaction with histones significantly shifted the absorption values of QDs and caused a reduction in intensity and redshift of the PL spectra. Furthermore, treating QDs with increasing concentrations of histones showed an increase in QD size and a change in zeta potential from −28.9 mV to 15 mV (upon 0.125 mg/mL histone treatment). Finally, FLIM measurements confirmed increased QD aggregation and reduction in PL lifetime with the addition of core histones [61]. These results revealed the strong affinity of QDs for histones. Similarly, a recent study by Le et al. also highlighted the ability of carboxylated CdSe/ZnS QDs to bind histones [62].

Conroy et al.’s study demonstrated the ability of negatively charged CdTe QDs to bind to core histones over other biopolymers, and while it helped to improve understanding on QD–protein interactions, the consequences of these interactions on gene expression and epigenetics were not explored. The study treated histones as isolated proteins and did not evaluate QD–histones in the context of nucleosome-bound DNA or chromatin organization, which limited the insights on epigenetic toxicology.

### 3.2. QD-Mediated Dysregulation of miRNA Expression

On the basis of these studies carried out by Choi et al. and Conroy et al., where QDs interact with histones and cause hypoacetylation, Li et al. examined how CdTe QDs affect the expression of miRNAs in NIH/3T3 fibroblast cells, focusing on miRNA biogenesis and the p53 signaling pathway [66]. The earlier study carried out by Choi et al. showed that certain proteins’ expression is regulated at the post-transcriptional level, which infers that miRNAs might play a role in this. MiRNAs and epigenetic mechanisms such as DNA methylation and histone modification are interconnected through a feedback loop. On the one hand, epigenetic mechanisms influence the expression and function of miRNAs and on the other hand, miRNAs modulate gene expression by regulating the expression of epigenetic enzymes such as DNA methyltransferases (DNMTs) and HDACs.

To examine and compare the changes in miRNA expression, Li et al. used SOLiD sequencing followed by Z-test analysis before and after QD treatment. The results showed a dose- and time-dependent response upon QD exposure, where 35 mature miRNAs were upregulated and 16 were downregulated. Additionally, the transcription levels of primary miRNAs (pri-miRNAs) and precursor miRNAs (pre-miRNAs) were also altered. However, the differentially expressed pri-miRNAs were distinct to that of pre-miRNAs, suggesting a dysregulation in miRNA processing [66]. This dysregulation can result in the downstream alteration in expression of their target mRNAs, causing variation in several other biological processes.

Similarly to the study carried out by Choi et al. [60], Li et al. also explored the expression levels of p53 as it is involved in the transcriptional regulation of miRNA genes involved in epigenetic control [66]. Consistent with Choi et al.’s findings, the mRNA levels of p53 remain unchanged but CdTe QD treatment significantly increased the protein levels of p53 and phosphorylated p53 (Table 2). This finding indicates the possibility of an indirect epigenetic effect because p53 are regulators of miRNAs that are associated with epigenetic enzymes like DNMTs and HDACs. However, while emphasizing the p53 pathway, the study did not explore other transcriptional or epigenetic regulators of miRNA expression such as Myc, NF-κB, and HDAC.

Altogether, this study highlights the dysregulation in miRNA expression at multiple stages of biogenesis caused by QD exposure. It also hints at miRNAs being both markers and mediators of QD-induced cytotoxicity. Although this paper mentions epigenetic aspects and references histone modifications, the study does not conduct any experiments related to histones, DNA methylation, or the chromatin state. The current literature tells us that chromatin accessibility and miRNA expression are intricately linked. Furthermore, the downstream effects of dysregulation in miRNA expression are also not explored. Additionally, while the study identifies several differentially expressed miRNAs (miR-29a, miR-93, miR-145, and miR-214) after QD treatment, it did not assess the impact of these differentially expressed miRNAs on cell viability or the cytotoxic nature of CdTe QDs. Therefore, these experiments are further needed to grasp the epigenetic risks posed by QDs.

Up until this point, the QDs being used have been mainly negatively charged, facilitating their interaction with histones. But recently in 2020, Sima et al. examined the effects of negatively charged carbon dots (nCDs) as well as positively charged carbon dots (pCDs) on human embryonic pulmonary fibroblasts (HEL 12469), comparing their whole-genome gene expression changes and gene-specific DNA methylation [63]. They treated HEL 12469 cells with a range of concentrations (0–500 µg/mL) of CDs (pCDs and nCDs) separately. pCD treatment resulted in a significant decrease in cell viability at concentrations of 50 µg/mL and above, whereas for the nCD treatment, there was no significant decrease in cell viability, even at 500 µg/mL.

The differentially expressed mRNAs were examined under four different exposure conditions. At 24 h, 50 µg/mL pCDs (24 h 50) altered 727 mRNAs (432 downregulated, 295 upregulated), while 100 µg/mL nCDs (24 h 100) altered 2266 (977 down, 1289 up). Additionally, pathway analysis linked pCDs to cell cycle, cholesterol biosynthesis, and mitochondrial function while nCDs impacted DNA repair, p53 signaling, translation, and cancer pathways.

Similar exposure conditions were utilized to study differentially expressed miRNAs as well where miRNA changes replicated the mRNA patterns, i.e., nCD treatment caused more differential expression. For miRNAs, 76 miRNAs were dysregulated after 24 h 50. Whereas for 24 h 100, 220 miRNAs were differentially expressed. The commonly affected pathways from miRNA–mRNA interactions included cancer signaling (Wnt, Ras, MAPK) (Figure 3), immune response, ECM interactions, and apoptosis (nCDs). In terms of DNA methylation, there was no differentially expressed methylated CpG region under the different exposure conditions. This was attributed to DNA methylation being a relatively slower process, and the longest exposure time used in the experiments was 24 h only, which might have been insufficient to cause any changes and detect epigenetic remodeling.

Overall, the study reiterated the importance of surface charge on quantum dots and how biological response is influenced by it. In terms of epigenetic evaluation, to the best of our knowledge, comparing between nQDs and pQDs, this study is the first of its kind. The study focuses on transcriptional changes, but it does so without evaluating protein expression and function to validate their transcriptional data. While the study also addresses DNA methylation as an epigenetic parameter, they utilized a gene specific approach instead of measuring the global 5-mC levels. Assessment of global DNA methylation in addition to the gene-specific approach would reveal a broader pattern across the whole genome. Furthermore, experiments should be carried out with an exposure duration longer than 72 h to be able to detect delayed epigenetic changes, if any.

### 3.3. QD-Induced Changes in DNA Methylation

In 2021, Ku et al. investigated whether graphene QDs (GQDs) influence mouse embryonic stem cell (mESC) pluripotency through epigenetic mechanisms, focusing on DNA methylation of pluripotency genes (*Sox2* and *Oct4*) [64]. A cytotoxicity investigation revealed that there was no significant decrease in cell viability upon 200 µg/mL GQD treatment for 48 h. In addition to cytotoxic studies, treatment with GQDs caused a delay in differentiation of mESCs into embryonic bodies (EBs) along with suppressing the transcriptional expression of biomarkers involved in all three germ layers (endoderm, mesoderm, and ectoderm). After the differentiation of mESCs into EBs in control cells, the transcriptional levels and protein expression of *Sox2*, *Nanog*, and *Oct4* significantly decreased.

However, GQD treatment (10 and 50 µg/mL) impaired the differentiation process by interfering with the silencing of *Sox2* and *Oct4*. Upon treatment, both concentrations slowed the decrease in the transcriptional and protein expression levels of *Sox2* and *Oct4*. Methylation analysis further revealed that 50 µg/mL GQD treatment inhibited CpG methylation of *Sox2* promoter (Figure 3), while *Oct4* methylation remained unaffected. This inhibition was accompanied with the significant decrease in mRNA levels of DNMTs (*Dnmt3a* and *Dnmt3b*) and increase in demethyltransferase (*Tet1*), thereby disrupting the normal DNA methylation process. Moreover, treatment with Bobcat339, a demethylase inhibitor, partially restored *Sox2* repression, confirming that the demethylation contributed to the impaired differentiation.

Altogether, this is the first study that demonstrates QDs’ ability to disrupt embryonic stem cell differentiation via an epigenetic process (DNA methylation of *Sox2* promoter). Following Sox-2 dysregulation, future studies should focus on tracking the downstream effects of this dysregulation. Additionally, global DNA methylation and histone modification patterns can be compared during differentiation of mESCs with and without GQD exposure.

Most recently in 2024, Cristian et al. utilized silicon (Si) QDs to evaluate its in vivo pulmonary and splenic toxicity in mice, focusing on tissue morphology, oxidative stress, apoptosis, and epigenetic changes by examining the global level of DNA methylation and histone modifications [65]. The study demonstrated that the SiQD treatment was generally well-tolerated at lower doses (1 mg/kg body weight). However, higher doses (100 mg/kg body weight) triggered tissue-specific response. At 100 mg/kg body weight SiQD administration, they identified that Superoxide dismutase (SOD) activity in lung tissues was more sensitive compared to splenic tissues as SOD activity decreased for lungs while it remained similar for spleen compared to the control. The reduction in SOD activity directly relates to increased oxidative stress which, in turn, can activate p53 [67]. p53 not only regulates cellular division and apoptosis but it also binds to DNMTs and maintains DNA methylation homeostasis [68]. As such, p53 expression was increased after 72 h SiQD treatment in lung samples, whereas for spleen samples, p53 expression decreased after 24 h but returned to near control levels after 72 h.

Using ELISA, they compared the global level of 5-mC in mice with and without SiQD treatment after 72 h. At 100 mg/kg body weight QD treatment, the 5-mC DNA in mice increased by 22% in lung and 41% in spleen samples compared to that of the control. These findings appear contrary to the observed p53 expression patterns, especially the lung samples, as p53 normally decreases global methylation by repressing DNMTs and activating TET enzymes. However, p53 has also been shown to promote methylation (Figure 3) at specific gene targets [69,70], which could help explain the elevated 5-mC DNA levels. In parallel, analysis of core histone, H4, under similar experimental conditions revealed the total H4 protein level decreased by 45% in lung samples while it increased by 10% in spleen samples compared to the control.

These findings highlight that the epigenetic responses are tissue-specific following SiQD exposure, and it would be interesting to see when these changes persist or revert in the long run. In addition to H4 histone changes, analyzing specific gene promoters, histone markers, and post-translational modification patterns (H3K27me3, H3K9ac) would strengthen the connection of SiQD changes and toxicity to epigenetics. It is also difficult to connect the epigenetic modifications to gene expression due to the absence of transcriptomic data. As such, including RNA sequencing, miRNA analysis, and biological tests to validate the changes would further help understand the consequences of the epigenetic changes.

**Table 2:** Overview of epigenetic changes caused by QDs.

Epigenetic Target	Cell Model	QDs Used	Key Aspects	Author, Year [Citation]
Histone modification	MCF-7	CdTe QDs	Pioneering study that discusses QDs’ impact on epigenetic processes (here, it causes histone hypoacetylation). QD-induced hypoacetylation reversed using TSA. Elevated levels of p-p53 after QD treatment.	Choi et al., 2008 [60]
Histone interaction	THP-1	CdTe QDs	Cellular tracking of QDs in nuclear areas where QDs show histone affinity.	Conroy et al., 2008 [61]
Gene (mRNA, miRNA) expression, DNA methylation	HEL 12469	CDs (nCDs and pCDs)	Charge-based comparison of CDs on epigenome where nCDs caused more changes in gene expression.	Sima et al., 2020 [63]
DNA methylation	mESCs	GQDs	QDs disrupt embryonic stem cell differentiation by altering DNA methylation of *Sox2* promoter.	Ku et al., 2021 [64]
DNA methylation, histone modification	Mouse (in vivo)	Si QDs	Epigenetic changes induced by QDs resulted in tissue-specific (lungs vs. spleen) responses.	Cristian et al., 2024 [65]
miRNA biogenesis	NIH/3T3	CdTe QDs	QD exposure results in dysregulation of miRNA expression. Protein levels of p53 and p-p53 elevated after QD treatment.	Li et al., 2011 [66]

## 4. Conclusions

While seemingly different, QDs and epigenetics are interlinked through their relatability and applications in biomedicine. Epigenetics elucidates how external environmental and lifestyle factors influence gene expression. Among these external factors, QDs are a class of nanoparticles that possess unique optical and chemical properties such that they are used as probes to visualize and detect epigenetic markers, providing the possibility of early and accurate diagnosis. Therefore, with increasing interest in understanding how nanomaterials influence gene regulation beyond genotoxicity, epigenetics is explored jointly with QDs in research for developing next-generation diagnostic tools.

Consequently, this review highlights the dual nature of QDs in epigenetics as powerful biosensing tools and as potential disruptors of gene regulation. While QDs’ diagnostic capabilities as biosensors are promising, there is also growing evidence that indicates QDs can disrupt epigenetic mechanisms by altering DNA methylation patterns, histone modification states, and miRNA expression. As such, the studies reviewed here are critically analyzed in the context of QDs and epigenetics. The studies dealing with the disruption of epigenetic mechanisms remain majorly limited in scope, dealing with short-term consequences, specific immortalized or cancerous cell lines, and focusing on isolated epigenetic mechanisms. Additionally, most studies report epigenetic changes but do not connect them to phenotypic outcomes. Without mentioning the functional consequences, it is hard to assess the biological significance caused by the epigenetic changes. There is also very limited exploration on whether these effects on epigenetics marks are passed down to the next generation.

While QDs’ diagnostic capabilities are promising, these gaps emphasize the need for a more comprehensive and multidisciplinary approach to fully understand the implications of QD exposure on epigenetics. Research should focus on longitudinal studies to explore delayed and heritable epigenetic changes. To help gain a better understanding of cell/tissue specific epigenetic responses, comparative analysis should be carried out on varied cell lines including non-cancerous human cells and primary cells as well as organoids that mimic in vivo physiology. Additionally, a multi-omics approach should be implemented to focus on connecting epigenetic changes to phenotypic outcomes including gene and protein expression, cellular behavior, and genetic predisposition for personalized medicine. Addressing these limitations will help gain a well-rounded understanding on QD–epigenetics interactions and, with the increasing applications of QDs in biomedicine diagnostics and therapeutics, also accentuate the necessity to incorporate epigenetic consequences in QD regulatory framework.

## Figures and Tables

**Table 1:** Studies that focus on QDs as probes for epigenetic changes.

Epigenetic Target	Detection Platform	QD Function	Key Aspects	Author, Year [Citation]
DNA methylation	QD-FRET system	QDs are donors that generate FRET signal to A647-labeled DNA.	Convenient, cost- and time-effective biosensor that enables highly sensitive early detection of DNA methylation in cancer tissues.	Ma et al., 2015 [35]
DNA methylation: Cytosine (C) and 5-methylcytosine (5-mC)	Nitrogen-doped CQD fluorescent sensor	Fluorescent label for detection.	Highly specific for detection of C and 5-mC in urine samples where fluorescent intensity is enhanced in presence of more C, in contrast to fluorescent quenching caused by 5-mC.	Thonghleung et al., 2023 [36]
Histone modification	QD-PEGDA hydrogel microparticles	QDs that emit red, green, and blue light are embedded in PEGDA hydrogel microparticles for color coding and detection using a single wavelength.	Highly sensitive simultaneous detection of multiple modified histones from the brain of cocaine exposed mice.	Yeom et al., 2016 [37]
miRNA expression	QD-FRET system	QDs are donors to induce Cy5 FRET signal	Highly sensitive and specific biosensor with possibility of being used as a multiplex detection system.	Zhang et al., 2011 [41]
miRNA expression	AuNPs@CdS QD FRET system	QDs are FRET donors measuring fluorescence quenching.	Enzyme-free and relatively simple biosensor for low-cost detection of let-7a miRNA.	Hosseini et al., 2025 [42]
miRNA expression	CdSe@CdS/TMC/Fe_3_O_4_ nanocomposites	QDs generated electrochemical signal proportional to concentration of let-7a miRNA.	PCR-free biosensor for detection of gastric cancer-specific miRNAs.	Daneshpour et al., 2018 [48]
Histone modification: Sirtuin 1 (SIRT1)	Cy5-labeled peptide substrate with a streptavidin-coated QD nanosensor	Peptides assemble on QD surface via streptavidin and induce FRET from QD to Cy5.	Highly sensitive novel deacetylation-activated QD sensor that achieves label-free detection of SIRT1 and its inhibitors.	Hu et al., 2021 [49]
DNA methylation	poly (β-cyclodextrin)—Ag/GQD nanocomposite	GQDs were hybridized with Ag nanoparticles as a substrate for electrode.	Detection of methylated and unmethylated sequences as well as sequences containing mismatch with very low detection limit.	Adampourezare et al., 2022 [50]
DNA methylation: 5-hydroxymethylcytosine (5-hmC)	QD-FRET system	biotin-/Cy5-labeled ssDNA assemble on QD surface via streptavidin and induce FRET for generation of Cy5 signal.	Very low detection limit of 5-hmC DNA in complex mixtures without adding reagents or specific antibiotics.	Wang et al., 2022 [51]
DNA methylation (5-mC) and RNA modification (N6-methyladenosine)	UiO-66@CdTe@AuNPs photoelectrochemical biosensor	CdTe QDs are used as light-absorbing semiconductors, resulting in efficient conversion of high photons and electrons that amplifies the sensor signal.	Robust and simultaneous detection of DNA methylation and RNA modification based on antibody-specific recognition.	Hu et al., 2025 [52]

## Data Availability

No new data were created or analyzed in this study. Data sharing is not applicable to this article.

## Abbreviations

The following abbreviations are used in this manuscript:

QDs	Quantum dots
miRNA	Micro-RNA
NPs	Nanoparticles
FRET	Fluorescence resonance energy transfer
A647	Alexa fluor-647
PCR	Polymerase chain reaction
LOD	Limit of detection
ROC	Receiver operating characteristic
MS-HRM	Methylation-sensitive high-resolution melting
PEGDA	Polyethylene glycol diacrylate
2Me-H3K9	Dimethylation of lysine 9 on histone H3
3Me-H3K9	Trimethylation of lysine 9 on histone H3
Ac-H3K9	Acetylation of lysine 9 on histone H3
SA-PE	Streptavidin-phycoerythrin
EXPAR	Exponential amplification reaction
HCR	Hybridization chain reaction
5-mC	5-methylcytosine
LC-MS	Liquid chromatography–mass spectrometry
ChIP-seq	Chromatin immunoprecipitation sequencing
MLPA	Multiplex ligation-dependent probe amplification
5-hmC	5-hydroxymethylcytosine
OxBS-seq	Oxidative bisulfite sequencing
TAB-seq	TET-assisted bisulfite sequencing
CdTe	Cadmium telluride
Ac-H3	Acetylated histone 3
HDAC	Histone deacetylase
TSA	Trichostatin A
WGBS	Whole-genome bisulfite sequencing
FLIM	Fluorescent lifetime imaging
PL	Photoluminescence
DNMTs	DNA methytransferases
Pri-miRNAs	Primary miRNAs
Pre-miRNAs	Precursor miRNAs
nCDs	Negatively charged carbon dots
pCDs	Positively charged carbon dots
GQDs	Graphene quantum dots
mESCs	Mouse embryonic stem cells
Si	Silicon
